# Tuning the Electronic Structure of Anatase Through Fluorination

**DOI:** 10.1038/srep11553

**Published:** 2015-06-26

**Authors:** Dario Corradini, Damien Dambournet, Mathieu Salanne

**Affiliations:** 1 Sorbonne Universités, UPMC Univ Paris 06, CNRS, UMR 8234, PHENIX, Paris, France

## Abstract

A highly fluorinated anatase lattice has been recently reported, providing a new class of materials whose general chemical formula is Ti_1−*x*_□_*x*_X_4*x*_O_2−4*x*_ (X^−^ = F^−^ or OH^−^). To characterise the complex structural features of the material and the different F environments, we here apply a computational screening procedure. After deriving a polarisable force—field from DFT simulations, we screen in a step-wise fashion a large number of possible configurations differing in the positioning of the titanium vacancies (□) and of the fluorine atoms. At each step only 10% of the configurations are retained. At the end of the screening procedure, a configuration is selected and simulated using DFT-based molecular dynamics. This allows us to analyse the atomic structure of the material, which is strongly disordered, leading to a strong decrease (by 0.8 eV) of the band gap compared to conventional anatase.

Titanium dioxide, TiO_2_, is a widely studied material. TiO_2_ has in fact several promising applications, for example in the fields of photocatalysis, green chemistry and energy storage^1^,^2^,^3^,^4^,^5^,^6^,^7^,^8^. Naturally occurring polymorphs of TiO_2_ include rutile, anatase and brookite. Recently, interest in the polymorphs of TiO_2_ has been sparked in particular by their possible application as anodic materials in Li ion batteries^9^,^10^,^11^,^12^,^13^,^14^. Fluorinated TiO_2_ has also been investigated^15^,^16^,^17^,^18^,^19^,^20^ since the presence of F in the compound might improve the sought characteristics of the material^17^ or stabilise the highly reactive {001} facets of the anatase crystal^19^,^21^. The nature of the fluorinated compound depends strongly on the fluorination technique employed^18^,^22^. So far, the stabilisation of fluorine within the anatase lattice of TiO_2_ has been poorly understood, probably because of the structural complexity of the fluorinated material.

Pure anatase is a tetragonal crystal, with *c* ≃ 2.5*a*, and its lattice is characterised by TiO_6_ octahedral units. Recently, a novel synthesis technique conducted in our laboratory^23^ has led to the preparation of a highly fluorinated anatase material in which fluoride or hydroxide anions replace the oxides in their lattice sites and the resulting charge deficiency is compensated by the formation of a cationic vacancy (□) every four substitutions. The material obtained has thus the general formula Ti_1−*x*_□_*x*_X_4*x*_O_2−4*x*_, where X^−^ = F^−^ or OH^−^ (the amount of F^−^ may vary depending on the synthesis conditions). Elemental analysis and synchrotron diffraction have revealed the existence of more than 20% cation vacancies. In fact the stoichiometric formula Ti_0.78_□_0.22_X_0.88_O_1.12_ has been assigned to the most fluorinated composition of the material. By using ^19^F NMR spectroscopy, it has also been possible to discern three different coordination modes for the F atoms: F − Ti_1_□_2_, F − Ti_2_□_1_ and F − Ti_3_, highlighting the complex structural arrangement present in the material.

Here we report the results of a computational study of the fully-fluorinated, hydroxide-free material (i.e. Ti_0.78_□_0.22_F_0.88_O_1.12_) performed in order to better characterise its structural features and the effect of fluorination on the electronic structure. The enormous number of possible structural arrangements of the vacancies and of the F atoms in the anatase structure render the problem untreatable directly by *ab initio* simulations. Therefore we apply a screening procedure on the possible configurations of the material, in the spirit of the emerging high—throughput techniques^24^,^25^,^26^, by using classical Molecular Dynamics (MD). Several force—fields have been previously proposed for pure TiO_2_^27^,^28^,^29^,^30^,^31^,^32^,^33^. In this work, we use a *polarisable* force—field valid for the pure phase^34^ as well as for the fluorinated material. We have extracted its parameters from Density Functional Theory (DFT) simulations, *via* a well-established force and dipole fitting procedure^35^,^36^. We have chosen to derive a new force—field instead of using an already available one for TiO_2_. This is motivated by the fact that we want the force—field to be compatible with O to F substitutions, as well as with other oxide species, e.g. SiO_2_, for future studies^34^. The details on the force—field employed are discussed in Supplementary Section S1, while an additional validation of the parameters involving fluorine atoms is presented in Supplementary Section S2.

In order to generate fluorinated samples starting from the pure TiO_2_ anatase, we apply a screening procedure, similar in spirit to what done by Wilmer *et al.* for metal-organic frameworks^37^ or by Coudert for zeolites^38^. At the fixed target composition Ti_0.78_□_0.22_F_0.88_O_1.12_, we consider samples containing F in all possible environments F − Ti_1_□_2_, F − Ti_2_□_1_, and F − Ti_3_, as suggested by NMR^23^. We leave the ratio of F in the different environments free to vary at random. The starting fluorinated structures are generated from the 4 × 4 × 2 pure anatase TiO_2_ structure^39^ (Ti_128_O_256_) leading to a system thus composed: Ti_100_□_28_F_112_O_144_. We generate these configurations by erasing 28 Ti ions at random with no constraints on the creation of adjacent vacancies and we randomly substitute 112 O with 112 F. We impose that all F and O must be attached to at least one Ti.

The screening procedure is then initiated. The protocol is as follows:

1. we perform single-point energy calculations on ≃1.5 ⋅ 10^5^ configurations; we then retain the ≃1.5 ⋅ 10^4^ configurations with the lowest energy for the following step.
2. we perform 0 K geometry optimisations of the atomic positions, keeping the length of the cell vectors fixed; we retain at maximum the 1.5 ⋅ 10^3^ configurations with the lowest energy for the following step.
3. we perform 0 K cell optimisations of both the atomic positions and the lengths of the cell vectors, while keeping the box angles fixed at *α* = *β* = *γ* = *π*/2; we retain at maximum the 1.5 ⋅ 10^2^ configurations with the lowest energy for the following step.
4. we temper the configurations performing 10 ps *NVT* runs at finite temperatures from *T*_1_ = 25 K to *T*_12_ = 300 K, every Δ*T* = 25 K. The 15 configurations with the lowest energy at *T*_12_ = 300 K are retained for the following step.
5. for the remaining samples, we perform a series of longer MD simulations at 300 K, first in the *NVT* and then in the *NPT* ensemble.
6. we then simulate the configuration with the lowest potential energy for 10 ps using using DFT—based molecular dynamics. We extract structural (bond length, fluorine environments) and electronic (density of states) characteristics of the material from this simulation.

Testing all the starting configurations in a generic, entirely *ab initio* based high-throughput procedure would be impossible. Generally, such studies involve static calculations only since performing *ab initio* MD simulations is computationally too expensive. Nevertheless, it is interesting to test whether our selected configurations, i.e., the 10 configurations remaining at the end of step 5) of the screening procedure would have also been selected if *ab initio* static calculations had been performed. To test this, we take their initial structures and perform a full DFT relaxation. Then we take the same number of random configurations from the starting pool of configurations. We find that the configurations given by the classical screening all have a lower final DFT energy than the ones taken at random. The results of this validation are shown in Supplementary Fig. S2.

Next, we analyse how the initial structural arrangements correlate with the energy of the configurations. The results are shown in Fig. 1. We see that the lowest energies (at 0 K) correlate with a higher fraction of F − Ti_2_□_1_. This is consistent with previous static DFT calculations performed on a system with only one vacancy and four O/F substitutions^23^, which showed that having the F closest to the vacancy stabilises the structure. In Fig. 1 we also report the initial energies of the best configurations given at the end by the screening procedure. We observe that these final screening configurations are found closer to the average F speciation values rather than at the highest F − Ti_2_□_1_ relative compositions. Some of them have strongly been stabilised during the procedure, showing the importance of taking into account relaxation and thermal effects (see also Supplementary Fig. S3).

In order to compare the structural properties of the material as found in the experiments with our simulations, we plot together the experimental x-ray structure factor *S*(*k*) and the one that we calculate from our trajectories using the Ashcroft—Langreth partial structure factors according to the formula:


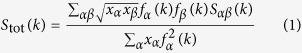


where *α*,*β* = Ti, O, F. *x*_*α*_ are the relative atomic concentrations of atoms of type *α*, *S*_*αβ*_(*k*) are the partial structure factors calculated from the simulation trajectories using


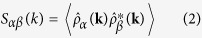


where the dynamic variable 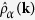 represents the Fourier component of the atomic density of type *α* atoms at wave vector **k**:


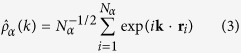


with **r**_*i*_ the position of atom *i*, and *N*_*α*_ the number of atoms of type *α* in the system. The angular brackets denote a thermal average, which was in practice evaluated as the time average over the whole simulation. Finally, *f*_*α*_(*k*) are the *k*-dependent atomic x—ray scattering factors. They are calculated using the analytic approximation:


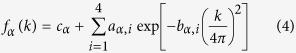


where the coefficients *a*_*α*,*i*_, *b*_*α*,*i*_ and *c*_*α*_ are taken from Ref. 40 for O^2−^ and from Ref. 41 for Ti^4+^ and F^−^. The structure factor calculated from the DFT-based molecular dynamics simulation performed on the final configuration is compared to the experimental signal in Fig. 2. The agreement between the two sets of data is good, taking into account that the experiments have been performed on nanoparticles, which leads to a strong broadening of the peaks, and that part of the fluoride ions are replaced by hydroxide groups. This may also affect the comparison, notwithstanding that F and OH are isoelectronic and thus their contribution to x-ray diffraction should not differ much if they occupy similar sites.

We also calculate the speciation of fluoride during the DFT-based molecular dynamics simulation. Contrarily to the initial configurations analysed in Fig. 1, the local relaxation of the F atoms (especially around the vacancies) leads to a wide distribution of Ti—F distances. It is therefore necessary to introduce a cutoff distance for assigning an environment to the F atoms. In Fig. 3 we show the time evolutions of the concentrations of F − Ti_1_□_2_, F − Ti_2_□_1_ and F − Ti_3_ for a cutoff of 2.7 Å which corresponds to the first minimum of the Ti—F radial distribution function. We observe that after 2 ps of simulation, the concentrations equilibrate around average values of 13/66/21% for F − Ti_1_□_2_, F − Ti_2_□_1_ and F − Ti_3_ respectively. This compares very well with the percentages measured by NMR in the experimental sample, i.e., 13/70/17%^23^. We can therefore conclude that the structure yielded by our screening procedure is realistic. This allows us to analyse it further in order to predict the material properties. We note that the F − Ti_2_□_1_ average concentration is larger than the corresponding fraction in the initial pool of configurations as shown in Fig. 1, because the fluorine atoms positions relax around the titanium vacancies during the first 2 ps of the simulation. However, no strong lattice rearrangements are observed, as can be seen from Supplementary Fig. S4.

The electronic structure is of particular interest for many applications, since TiO_2_-based materials are widely used in photocatalysis. We have therefore calculated the electronic density of states of fluorinated anatase on a series of snapshots extracted from our DFT-based molecular dynamics simulation, and compared it with the case of pure TiO_2_ anatase. We have used the hybrid functional HSE06^42^,^43^ for these calculations. In agreement with previous works^44^, we see in Fig. 4 that the valence band edge of pure TiO_2_ anatase is dominated by O 2*p*, and the conduction band edge is formed by Ti 3*d*. The band gap is much narrower in Ti_0.78_□_0.22_O_1.12_F_0.88_, by 0.8 eV. Unlike the case of conventional doping with heteroatoms^45^, the additional 2*p* states associated with fluoride ions do not locate at the top of the valence band, but rather at its bottom. The strong narrowing of the band gap is therefore due to the different structure of the material. In a previous study on TiO_2_ nanocrystals, Chen *et al.* have shown that, due to the presence of structural disorder, their materials exhibit a band gap substantially smaller than the one of pure bulk materials^46^. It is very likely that similar effects are at play here.

The structural disorder is apparently at the origin of these strong electronic structure changes. To test this idea, in Fig. 5 we show the partial radial distribution functions at ambient conditions *g*_*αβ*_(*r*) for the simulated pure anatase TiO_2_ and for the fluorinated anatase configuration selected by the screening procedure. The effect of the disorder introduced by the vacancies is immediately evident looking at the *g*_Ti−Ti_(*r*) and at the *g*_O−O_(*r*). The *g*_Ti−O_(*r*) structure seems to be conserved to a large extent at least for the first two shells, although the presence of vacancies induces a shortening of the first neighbour distance. The effect of disorder is also clear when looking for example at the region in between the first two peaks. We also observe that on the one hand, *g*_Ti−O_(*r*) and *g*_Ti−F_(*r*) are very similar, and so are the *g*_O−O_(*r*), *g*_F−F_(*r*) and *g*_O−F_(*r*). This confirms that the fluorine atoms substitute the oxygen ones inside the anatase structure.

In conclusion, in order to characterise fluorinated anatase Ti_0.78_□_0.22_F_0.88_O_1.12_, we have developed a screening procedure employing a polarisable force—field. It has allowed us (i) to select the best configurations starting from a very large pool (hundreds of thousands) of possible configurations; (ii) to reproduce the experimental structure, (iii) to study details of the partial atomic and electronic structure using DFT-based molecular dynamics. Our results show that fluorinated anatase has a highly disordered structure, which results in a lower band gap, by 0.8 eV, compared to conventional anatase. Therefore we conclude that fluorination appears as a very promising route for tuning material properties. This may be exploited for several applications, for example photocatalysis.

## Methods

We have performed the classical simulations using the software CP2K (single point calculations/geometry/cell optimisations, i.e., steps 1) to 3) of the screening procedure) and the in-house simulation software PIMAIM (molecular dynamics simulations, step 4) and 5) of the screening procedure). We have cut off the short—range interactions at half the norm of the shortest box vector (or less in *NPT* runs). The time step for the integration of the equations of motion has been set to 1 fs.

The DFT-based MD simulation has also been performed using the software CP2K^47^, using the Quickstep algorithm. We have used the GGA PBE^48^ exchange-correlation functional and we have employed the DZVP-MOLOPT-SR-GTH basis set^49^. Moreover, we have used the Goedecker-Teter-Hutter^50^ pseudo-potentials; for Ti atoms, the electronic orbitals explicitly represented are 3*s*^2^3*p*^6^3*d*^2^4*s*^2^, for O atoms 2*s*^2^2*p*^4^ and for F atoms 2*s*^2^2*p*^5^. We have set a plane wave cut-off of 400 Ry. We have added dispersive interactions through the use of the DFT-D3 correction^51^, with a cutoff radius of 30 Å. We have accumulated the trajectory for 10 ps, with the simulations time step being 0.5 fs. We have conducted the simulation in the *NVT* ensemble with a target temperature of 300 K. We have calculated the electronic density of states on a series of snapshot extracted from the trajectory, using the HSE06 functional^42^,^43^.

## Additional Information

**How to cite this article**: Corradini, D. *et al.* Tuning the Electronic Structure of Anatase Through Fluorination. *Sci. Rep.* **5**, 11553; doi: 10.1038/srep11553 (2015).

## Supplementary Material

Supplementary Information

## Figures and Tables

**Figure 1:**
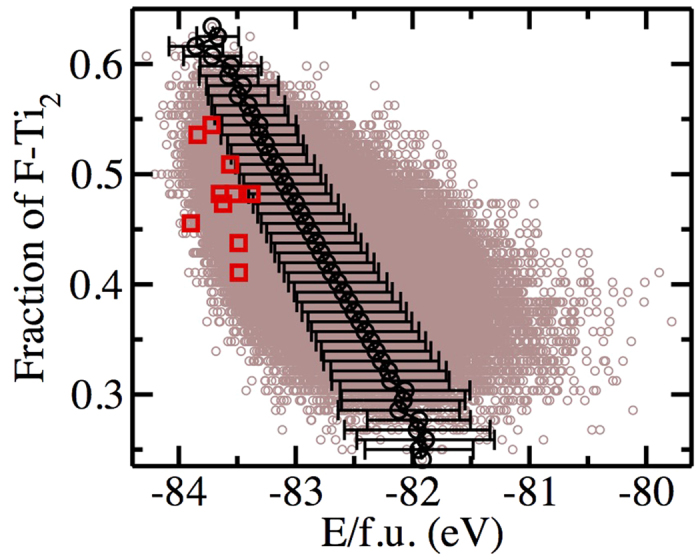
Energy—structure relation. Fraction of F − Ti_2_□_1_ vs. energy of the configuration for the initial configurations at 0 K. The points are represented as small brown circles. For each different value of the fraction of F − Ti_2_□_1_ we calculate the mean (black circles) and the standard deviation (black bars) of the corresponding energies. We also report the values assumed at this stage by the configurations run at the final screening step (red squares).

**Figure 2:**
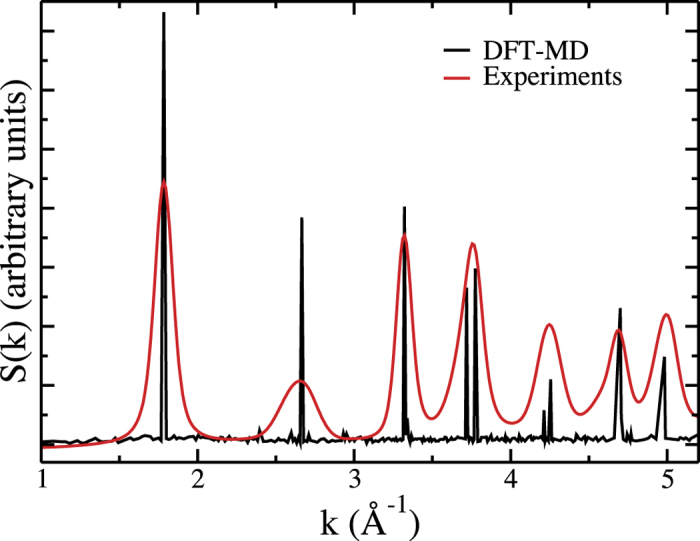
Structure Factor. Comparison of the structure factor *S*(*k*) at ambient conditions measured in experiments (red line) and calculated from a DFT-based molecular dynamics simulation performed on the configuration selected by the screening procedure (black line).

**Figure 3:**
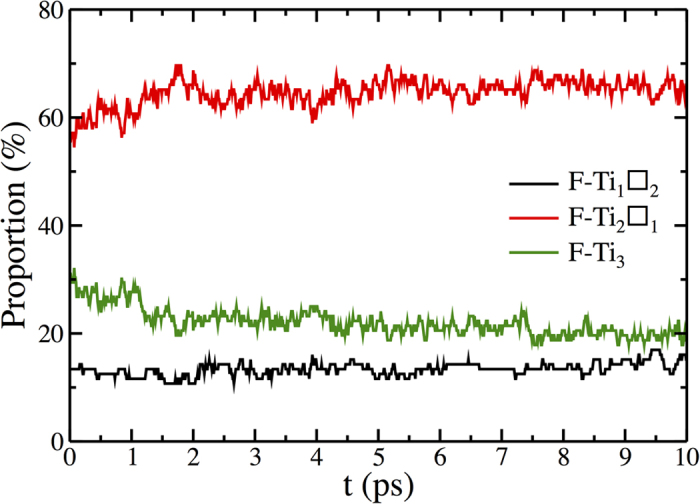
Fluorine coordination number. Evolution of the proportion of each coordination mode for the fluorine during the DFT—based MD simulation. The cutoff distance for defining Ti—F neighbour atoms is set to 2.7 Å.

**Figure 4:**
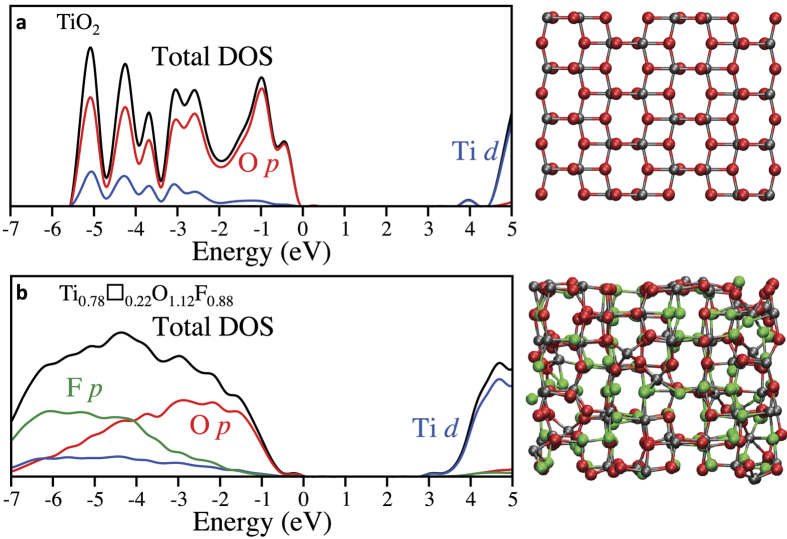
Electronic density of states. Comparison of the total and ion-decomposed density of states of TiO_2_ (**a**) and Ti_0.78_□_0.22_O_1.12_F_0.88_ (**b**) calculated using the HSE06 functional. Only the main contributions from the decomposition are shown. The plot for Ti_0.78_□_0.22_O_1.12_F_0.88_ corresponds to the final snapshot of the simulation, shown on the right side. No significant changes have been observed for other snapshots, see Supplementary Fig. S5.

**Figure 5:**
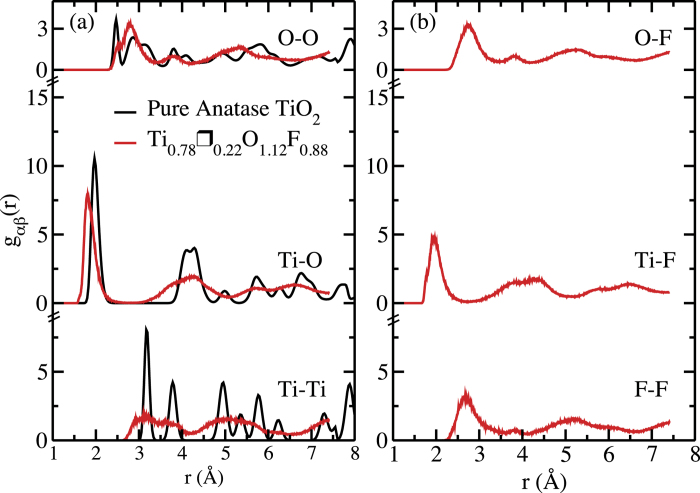
Radial distribution functions. Partial radial distribution functions *g*_*αβ*_(*r*) at ambient conditions calculated from a classical MD simulation of pure TiO_2_ anatase (black) and from a DFT-based simulation of the configuration selected by the screening procedure (red).
