# Supplementary material for: Tuning the Electronic Structure of Anatase Through Fluorination
Source: Sci Rep. 2015 Jun 26;5:11553. doi: 10.1038/srep11553 (PMC5387870; doi:10.1038/srep11553)
Supplement: Supplementary Information [file srep11553-s1.pdf]

Supplementary information for "Tuning the Electronic Structure of Anatase Through Fluorination"

Dario Corradini,* Damien Dambournet, and Mathieu Salanne†

Sorbonne Universités, UPMC Univ Paris 06,

CNRS, UMR 8234, PHENIX, Paris, France

(Dated: April 22, 2015)

* Correspondence and request for materials should be addressed to D. C. (dario.corradini@ens.fr); Current address: Laboratoire PASTEUR, UMR 8640 ENS–CNRS–UPMC Paris 6, Département de Chimie, École Normale Supérieure, 75005 Paris, France.

† Correspondence and request for materials should be addressed to M. S. (mathieu.salanne@upmc.fr).

S1. CLASSICAL MODEL

We describe the interaction potential between the ions by a classical polarisable force–field whose parameters we derive from *ab initio* DFT simulations. Details on how to extract the parameters of the classical force–field from DFT simulations are reported in earlier works (see Ref. [34–36] in the main text). In particular, the detailed procedure used for the pure phases of TiO_2 together with its validation are reported in Ref. [34] (of the main text) and are not repeated here. The force–field parameters for the fluoride ions are obtained in an analogous fashion. We do report here in the following the analytic form of the classical force–field that we use, together with its parameters.

A. Polarisable ion model (PIM)

The repulsive and dispersive terms of the interactions are taken into account using the the Born–Mayer–Huggins (BMH) form of the interaction potential:

$$V_{\mathrm{BMH}} = \sum_{i,j>i} A_{ij} e^{-a_{ij} r_{ij}} - f_6^{ij}(r_{ij}) \frac{C_6^{ij}}{r_{ij}^6} - f_8^{ij}(r_{ij}) \frac{C_8^{ij}}{r_{ij}^8} \,. \tag{S1}$$

The damping functions are Tang-Toennies functions of the form

$$f_n^{ij}(r_{ij}) = 1 - e^{b_D^{ij} r_{ij}} \sum_{k=0}^{n} \frac{(b_D^{ij} r_{ij})^k}{k!} \,. \tag{S2}$$

When performing molecular dynamics simulations, we add a Gaussian term in the Ti–O and Ti–F interactions that acts as a steep repulsive wall and accounts for the oxide/fluoride anion hard core:

$$V_{\mathrm{Gaussian}} = \sum_{i \in \mathrm{O,F}, j \in \mathrm{Ti}} B_{ij} e^{-d_{ij} r_{ij}^2} \,. \tag{S3}$$

This extra term is used in cases where the ions are strongly polarised to avoid instability problems at very small anion–cation separations.

For the Coulombic part of the interaction potential,

$$V_{\mathrm{Coulomb}} = \sum_{i,j>i} \frac{q_i q_j}{r_{ij}} \,, \tag{S4}$$

the formal charges for the ionic species are used, $-2e$ for O ions, $-e$ for F ions and $+4e$ for Ti ions. The many–body electrostatic interactions are described by the induced dipoles $\boldsymbol{\mu}_i$, obtained at each MD step minimising the polarisation energy

$$V_{\rm pol} = \sum_i \frac{1}{2\alpha_i}|\boldsymbol{\mu}_i|^2 + \sum_{i,j>i} \left[\left(q^i \mu^j_\alpha g^{ij}(r_{ij}) - q^j \mu^i_\alpha g^{ji}(r_{ij}) \right) T^\alpha_{ij} - \mu^i_\alpha \mu^j_\beta T^{\alpha\beta}_{ij} \right] \tag{S5}$$

where the Einstein summation convention is assumed, α_i is the atomic polarisability and T are the multipole interaction tensors. The damping function $g_{ij}(r_{ij})$ is of the Tang-Toennies form

$$g_{ij}(r_{ij}) = 1 - c_{ij} e^{-b_{ij} r_{ij}} \sum_{k=0}^{4} \frac{(b_{ij} r_{ij})^k}{k!} \, . \tag{S6}$$

B. Parameterisation

The repulsion and polarisation parameters of the force–field have been fitted in order to reproduce the forces and dipoles extracted from DFT calculations, using a well-established procedure, see Ref. [34] in the main text. In the present case, we obtain final χ^2 values of 0.16 and 0.37 for the fits of dipoles and forces, respectively. Such values are similar to the ones obtained in our recent work on other oxide materials, i.e. rare earth doped ceria, for example [S1]. The dispersion interactions are not taken into account in a proper way in the DFT calculations we have performed. The corresponding parameters have not been fitted, instead they have been taken from our previous works on fluorides and oxides, see Ref. [35] in the main text. Finally, the Gaussian term parameters have been chosen following Marrocchelli *et al.* [S2]. The obtained parameters are reported in Table **S1** for the BMH part of the force–field and in Table **S2** for the polarisation part.

Supplementary Table S1. **BMH parameters.** Parameters of the BMH potential extracted from DFT simulations.

Atom Pair	A_{ij} (Ha)	a_{ij} (Å^{-1})	B_{ij} (Ha)	d_{ij} (Å^{-2})	C_6^{ij} (Ha Å^6)	C_8^{ij} (Ha Å^8)	b_D^{ij} (Å^{-1})
O–O	290.4	4.54668	–	–	0.48309	2.61949	2.64562
O–F	278.4	4.71487	–	–	0.39890	1.55438	3.11805
O–Ti	43.0	2.86431	50,000	6.4279	–	–	–
F–F	282.3	4.61849	–	–	0.32938	0.922357	3.59048
F–Ti	28.3	3.13082	50,000	6.4279	–	–	–
Ti–Ti	1.0	9.44863	–	–	–	–	–

Supplementary Table S2. **Polarisation parameters.** Parameters of the polarisation part of the interaction potential extracted from DFT simulations.

Atom / Atom Pair	α (Å^3)	b_{ij} (Å^{-1})	c_{ij}
O	1.59150		
O–O		4.74888	2.227
O–F		–	–
O–Ti		3.90122	2.13327
F	1.16458		
F–O		–	–
F–F		–	–
F–Ti		4.16887	2.90678
Ti	0.20442		
Ti–O		3.90122	-1.90330
Ti–F		4.16887	-2.66057
Ti–Ti		–	–

S2. COMPARISON BETWEEN AB INITIO AND CLASSICAL SIMULATIONS

We assess the behaviour of our force–field in the fluorinated samples, by comparing the results of classical and DFT calculations. For simplicity, we consider the case of one single fluorination, as in Ref. [23] in the main text. We consider here the system $Ti_{127}\square_1F_4O_{252}$ and we distribute the F atoms either at random positions in the lattice (where they substitute O atoms) or at positions neighbouring the cationic vacancy. The latter correspond to 2–coordinated F, i.e. $F-Ti_2\square_1$. We consider the cases where 0, 1, 2, 3 or 4 F are neighbouring the vacancy and have therefore a $F-Ti_2\square_1$ environment, with the remaining F having the $F-Ti_3$ environment. We compare the energy calculated in 0 K cell optimisations, using either DFT or our force field. The comparison for the energies is shown in Fig. **S1**. We see that our classic force-field is able to closely reproduce the decrease in energy with the increase in the number of $F-Ti_2\square_1$, observed by DFT calculations (see also Ref. [23] in the main text).

Finally in Fig. **S2** we show the comparison between the DFT energies of the configurations selected by the screening procedures and the same number of configurations taken at random from the initial pool of configurations of the $Ti_{100}\square_{28}F_{112}O_{144}$, before (panel a) and after (panel b) relaxation of the atomic positions and of the cell dimensions.

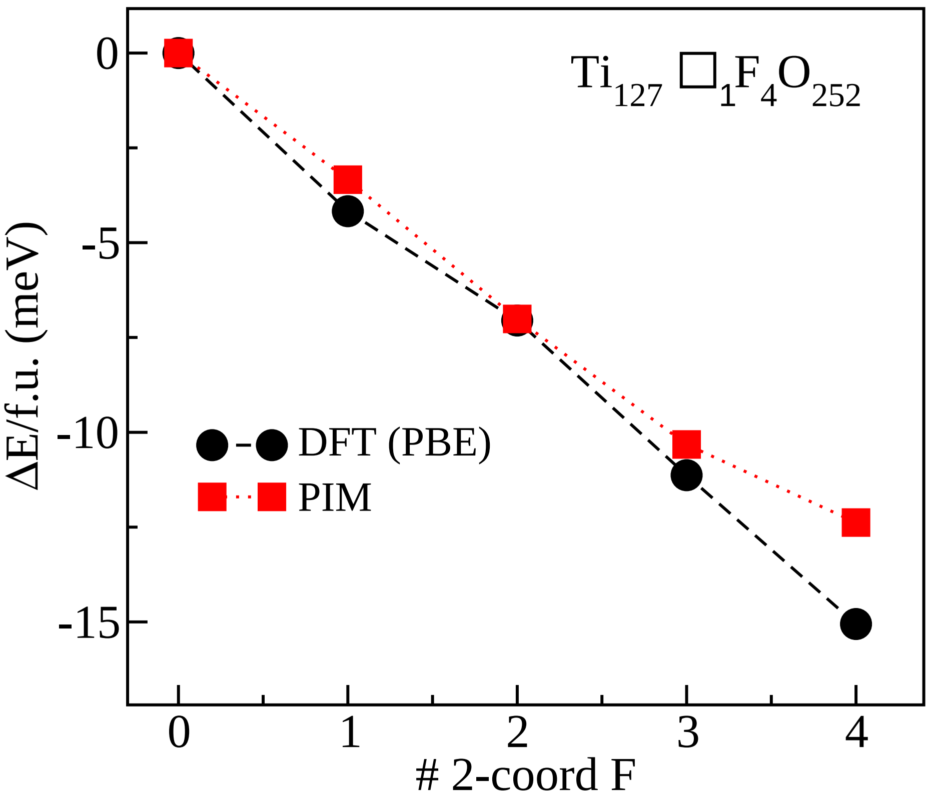

Supplementary Figure S1. **Comparison between DFT and classical potential.** Difference in energy between the case where all F atoms in $Ti_{127}\square_1F_4O_{252}$ have environment $F - Ti_3$ and the cases where F is progressively added in positions neighbouring the vacancies and has thus $F - Ti_2\square_1$ environment. We have calculated this quantity by DFT (circles) and by our classic force–field (squares).

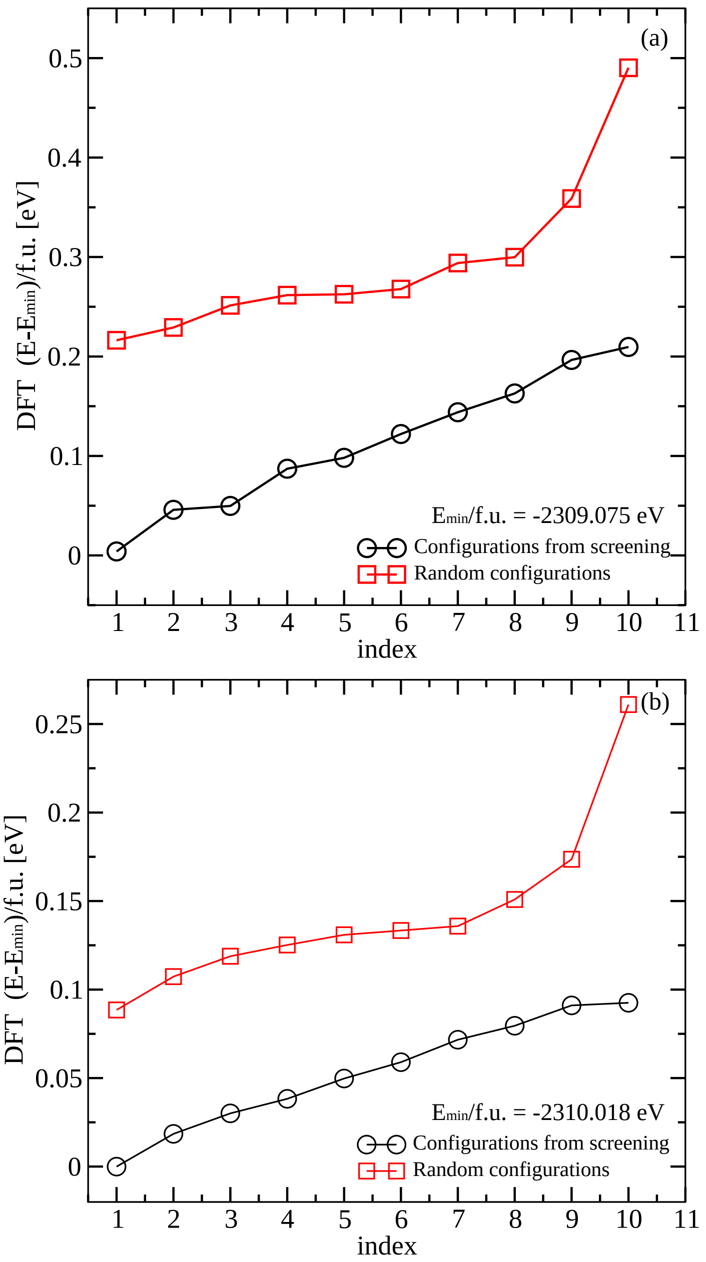

Supplementary Figure S2. **DFT energies of the configurations selected by the screening.** For the configurations left at the end of the screening procedure, we take their initial structures and calculate their DFT energy (black circles) before (a) and after relaxation (b). Those are compared with the DFT energies of the same number of configurations taken at random (red squares) from the initial pools of configuration. The values of the energies are plotted relative to the lowest DFT energy configuration. Panel (a) shows the results obtained before the DFT relaxation, panel (b) shows the results obtained after the DFT relaxation.

S3. SCREENING PROCEDURE

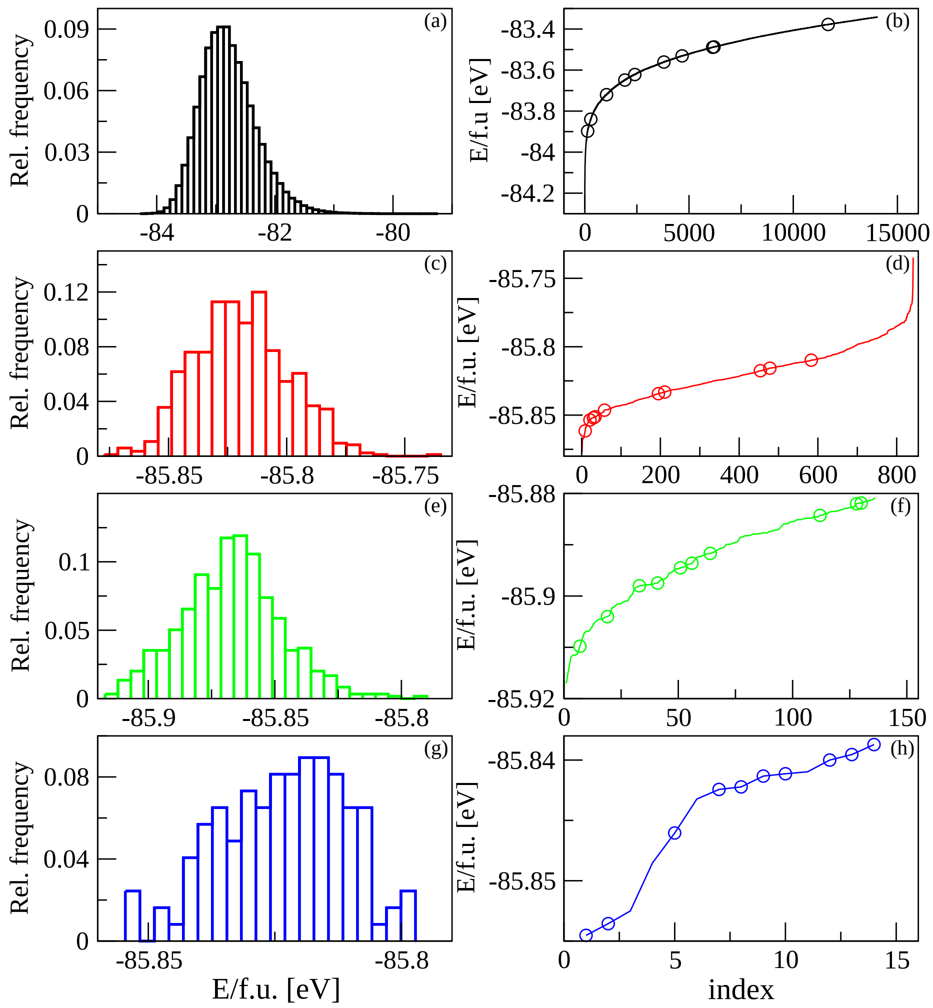

Supplementary Figure S3. **Energy distributions at screening steps 1) to 4).** The panels on the left show the relative frequency histograms of the energies calculated for all the configurations tested at each step, while the panels on the right show the sorted energies of the configurations retained after each step. The energies shown are: (a,b) at 0 K for the starting unrelaxed structures; (c,d) at 0 K after the optimization of the atomic positions; (e,f) at 0 K after the optimization of the atomic positions and cell vector lengths; (g,h) at 300 K after the tempering from 25 to 300 K. The open circles in panels (b,d,f,h) indicate the energy of the configurations left at the end of the screening procedure at each previous step. Note that the number of retained configurations can be less than the target one (see main text). This is due to “crashed” unstable configurations that are eliminated from the pool.

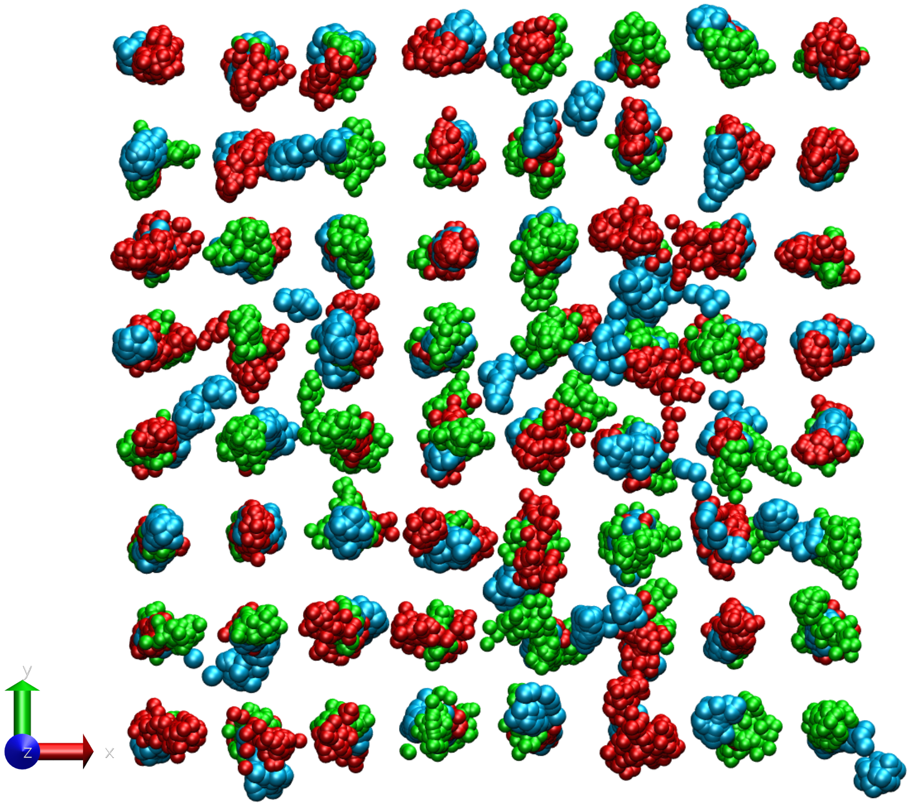

Supplementary Figure S4. **Positions of the atoms along the DFT–based MD simulation.** The positions of the Ti (blue), O (red) and F (green) atoms are shown every 10 steps of the simulation. Although structural relaxation close to the vacancies is observed, there is no major lattice rearrangement.

S4. $\mathbf{TI_{0.78}\square_{0.22}O_{1.12}F_{0.88}}$ DENSITY OF STATES

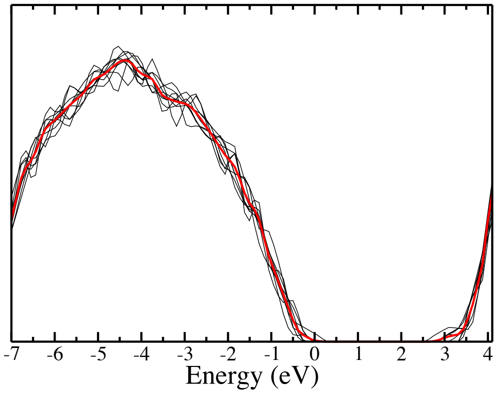

Supplementary Figure S5. **Density of states for several $\mathbf{Ti_{0.78}\square_{0.22}O_{1.12}F_{0.88}}$ configurations.** The density of states has been calculated for 10 configurations extracted from the DFT–based molecular dynamics trajectory. The results are shown as thin black lines while their average is shown using a thick red line.

S5. CP2K INPUT FILE

We here report an example of CP2K input file, prepared for running the system $Ti_{127}\Box_1F_4O_{252}$ in the NVT ensemble at $T = 300$ K, using our classical force–field.

```
&GLOBAL
 PROJECT 1fn-loc-npt
 RUN_TYPE MD
 PRINT_LEVEL LOW
 WALLTIME 50000
 &PRINT
  &EACH
   MD 500
  &END EACH
 &END PRINT
&END GLOBAL

&MOTION
 &MD
  &THERMOSTAT
   &NOSE
    LENGTH 3
    YOSHIDA 3
    TIMECON 1000.0
    MTS 2
   &END NOSE
  &END THERMOSTAT
 ENSEMBLE NVT
 STEPS 100000
 TIMESTEP 1
 TEMPERATURE 300.0
 &PRINT
  &PROGRAM_RUN_INFO
   &EACH
    MD 500
   &END EACH
  &END PROGRAM_RUN_INFO
  &ENERGY
   &EACH
    MD 500
   &END EACH
  &END ENERGY
 &END PRINT
 &END MD

 &PRINT
  &TRAJECTORY
   &EACH
    MD 5000
   &END EACH
  &END TRAJECTORY
  &RESTART_HISTORY
   &EACH
    MD 10000
   &END EACH
  &END RESTART_HISTORY
  &RESTART
   BACKUP_COPIES 1
  &END RESTART
  &END PRINT

&END MOTION

&FORCE_EVAL
 METHOD FIST
 &MM
  &FORCEFIELD
   &SPLINE
    EMAX_SPLINE 8.0
    RCUT_NB 7.541
   &END SPLINE
  &NONBONDED
   &BMHFTD
    atoms O O
    A 290.4
    B 4.54668
    C 0.483092
    D 2.61949
    BD 2.64562
   &END BMHFTD
   &BMHFTD
    atoms O F
    A 278.4
    B 4.71487
    C 0.3989
    D 1.55438
    BD 3.11805
   &END BMHFTD
   &BMHFTD
    atoms O Ti
    A 43.0004
    B 2.86431
    C 0.0
    D 0.0
    BD 0.0
   &END BMHFTD
   &BMHFTD
    atoms F F
    A 282.3
    B 4.61849
    C 0.32938
    D 0.922357
    BD 3.59048
   &END BMHFTD
   &BMHFTD
    atoms F Ti
    A 28.3129
    B 3.13082
    C 0.0
```

```
  D 0.0
  BD 0.0
 &END BMHFTD
 &BMHFTD
  atoms Ti Ti
  A 1.0
  B 9.44863
  C 0.0
  D 0.0
  BD 0.0
 &END BMHFTD
&END NONBONDED
&CHARGE
 atom O
 CHARGE -2.0000
&END CHARGE
&CHARGE
 atom F
 CHARGE -1.000
&END CHARGE
&CHARGE
 atom Ti
 CHARGE 4.000
&END CHARGE
&DIPOLE
 atom O
 APOL 1.59150
 &DAMPING
  TYPE Tang-Toennies
  ATOM O
  BIJ 4.74888
  ORDER 4
  CIJ 2.227
 &END DAMPING
 &DAMPING
  TYPE Tang-Toennies
  ATOM F
  BIJ 0.0
  ORDER 4
  CIJ 0.0
 &END DAMPING
 &DAMPING
  TYPE Tang-Toennies
  ATOM Ti
  BIJ 3.90122
  ORDER 4
  CIJ 2.13327
 &END DAMPING
&END DIPOLE
&DIPOLE
 atom F
 APOL 1.16458
 &DAMPING
  TYPE Tang-Toennies
  ATOM O
  BIJ 0.0
  ORDER 4
  CIJ 0.0
 &END DAMPING
 &DAMPING
  TYPE Tang-Toennies
  ATOM F
  BIJ 0.0
  ORDER 4
  CIJ 0.0
 &END DAMPING
 &DAMPING
  TYPE Tang-Toennies
  ATOM Ti
  BIJ 4.16887
  ORDER 4
  CIJ 2.90678
 &END DAMPING
&END DIPOLE
&DIPOLE
 atom Ti
 APOL 0.20442
  &DAMPING
   TYPE Tang-Toennies
   ATOM O
   BIJ 3.90122
   ORDER 4
   CIJ -1.90330
  &END DAMPING
  &DAMPING
   TYPE Tang-Toennies
   ATOM F
   BIJ 4.16887
   ORDER 4
   CIJ -2.66057
  &END DAMPING
  &DAMPING
   TYPE Tang-Toennies
   ATOM Ti
   BIJ 0.0
   ORDER 4
   CIJ 0.0
  &END DAMPING
 &END DIPOLE
&END FORCEFIELD
&POISSON
 &EWALD
  EWALD_TYPE EWALD
  EWALD_ACCURACY 1.0e-6
  ALPHA 0.39297
  RCUT 7.541
  GMAX  19
  O_SPLINE 6
  &MULTIPOLES T
   MAX_MULTIPOLE_EXPANSION DIPOLE
   POL_SCF CONJUGATE_GRADIENT
   EPS_POL 1.0e-6
   MAX_IPOL_ITER 100
  &END MULTIPOLES
 &END EWALD
&END POISSON
&PRINT
 &ITER_INFO
```

```
    &EACH
     MD 500
    &END EACH
   &END ITER_INFO
  &END PRINT
 &END MM
  &SUBSYS
    &CELL
      ABC 15.082   15.100   20.431
     PERIODIC XYZ
    &END CELL
    &COORD
Ti          0.0092526897        2.8105961256        1.2817567910
Ti          0.0506841050        2.8142375601       11.4965161278
Ti          0.0373622332        6.5721801714        1.2709321758
Ti          0.0686762730        6.5665219023       11.4946324231
Ti          0.0562138844       10.3752296101        1.2665654437
Ti          0.0930430310       10.3904422990       11.4782100093
Ti          0.0549241715       14.1400567348        1.2665611962
Ti          0.0936365683       14.1524822380       11.5041248161
Ti          3.7759452413        2.7912937424        1.2688107899
Ti          3.8063797628        2.7956184571       11.4642638411
Ti          3.8078281440        6.6046747939        1.2696113779
Ti          3.8492414420        6.6184808818       11.4971070498
Ti          3.7974199211       10.3708698515        1.2688784727
Ti          4.0203828276       10.3677305287       11.5495142728
Ti          3.8655421361       14.1362671796        1.2900093286
Ti          3.9310964920       14.1262510213       11.5145891174
Ti          7.5491134334        2.8313499507        1.2719267327
Ti          7.5681863082        2.8382965342       11.4772300865
Ti          7.5368925139        6.5978464134        1.2781345496
Ti          7.5546748184        6.6174658832       11.5111042655
Ti          7.6040157639       10.3627812967        1.2805033676
Ti          7.4622388553       10.3668239102       11.5501643194
Ti          7.6175012880       14.1114312193        1.2650385392
Ti          7.6209604718       14.1130553468       11.4826702741
Ti         11.2875516032        2.8265300096        1.2750800406
Ti         11.3235793462        2.8352807892       11.5014763035
Ti         11.3484903199        6.5950075403        1.2739718867
Ti         11.3781032837        6.6029488983       11.4994060714
Ti         11.3726570777       10.3438744021        1.2627720004
Ti         11.3869501154       10.3461423554       11.4690935325
Ti         11.3948366064       14.1469191252        1.2706971376
Ti         11.4054648836       14.1624354118       11.5083237067
Ti          1.8955489882        0.9119266960        6.3887525432
Ti          1.8978237343        0.9133769789       16.5865526041
Ti          1.9257135506        4.6895911922        6.3785533854
Ti          1.9404118027        4.7089906429       16.6043602590
Ti          1.9343509155        8.4920662432        6.3790619262
Ti          1.9518346955        8.4999711634       16.5905279568
Ti          1.9525165596       12.2628866292        6.3944507948
Ti          1.9926244726       12.2501341949       16.5875104054
Ti          5.6656627583        0.9131930570        6.3865663437
Ti          5.6901942967        0.9481698804       16.6330334148
Ti          5.6745443041        4.7141749439        6.3817441594
Ti          5.6728571349        4.7060333573       16.6151178373
Ti          5.6902441722        8.4904488086        6.3989824939
Ti          5.7153330731        8.6552556067       16.5614923347
Ti          5.7379715177       12.2412941842        6.3973018808
Ti          5.7423884378       12.0693424778       16.5737394374
Ti          9.4163802659        0.9350747908        6.3775979904
Ti          9.4189961791        0.9328323469       16.5879520691
Ti          9.4321743134        4.7199806153        6.3924056704
Ti          9.4612587123        4.7409793512       16.5808044328
Ti          9.4762792944        8.4699692502        6.3887627110
Ti          9.4708341809        8.4964066142       16.5917323933
Ti          9.5120319609       12.2363807806        6.3787708348
Ti          9.5229186076       12.2437664886       16.6039733162
Ti         13.1755604919        0.9384059638        6.3897255094
Ti         13.1948430181        0.9502852356       16.5943500922
Ti         13.2282803150        4.6946721385        6.3859403535
Ti         13.2413926828        4.7075475043       16.5935058981
Ti         13.2653352736        8.4621554862        6.3840386973
Ti         13.2856875386        8.4838322335       16.6183482562
Ti         13.2613652772       12.2639923032        6.3832808596
Ti         13.2723944261       12.2572329389       16.6016691772
Ti          1.8726842508        2.8277145271        3.8381380587
Ti          1.9277061239        2.8560282353       14.0457425718
Ti          1.9037779579        6.5985225931        3.8219885884
Ti          1.9500277893        6.5995357070       14.0377142884
Ti          1.9505542997       10.3542152293        3.8309291523
Ti          1.9188210756       10.3586744344       13.9999859258
Ti          1.9606396537       14.1244640472        3.8294548938
Ti          2.0081827508       14.1401083529       14.0407335203
Ti          5.6451516258        2.8211456330        3.8251639215
Ti          5.6771534617        2.8233289488       14.0137900642
Ti          5.6939306575        6.5802023077        3.8398661875
Ti          5.7110707339        6.5163552861       14.0922412885
Ti          5.7065685497       10.3536592289        3.8328950365
Ti          5.7259910744       14.1529049523        3.8440195023
Ti          5.7839305475       14.2542023809       14.1071208952
Ti          9.4414783466        2.8054100029        3.8378804521
Ti          9.4601022918        2.8089556560       14.0435886792
Ti          9.4559310186        6.5808125184        3.8289539910
Ti          9.4828400504        6.6110676822       14.0401584228
Ti          9.4686206627       10.3787434428        3.8322819245
Ti          9.5764227300       10.3957776869       14.0042592707
Ti          9.4902917028       14.1449715650        3.8183244626
Ti          9.4994241638       14.1413719375       14.0343092357
Ti         13.1963868466        2.8028921853        3.8330370903
Ti         13.2349776080        2.8221974625       14.0457430133
Ti         13.2086296573        6.6062484093        3.8343715458
Ti         13.2553567868        6.6389670489       14.0553270443
Ti         13.2378534778       10.3725492480        3.8225087699
Ti         13.2727412239       10.3668247532       14.0490364276
Ti         13.2906480096       14.1262907986        3.8330226768
Ti         13.3149589939       14.1295796332       14.0592257214
Ti          0.0066710895        0.9204443731        8.9458796274
Ti          0.0148939416        0.8884050857       19.1569922011
Ti          0.0146594993        4.7197127478        8.9348798870
Ti          0.0132231931        4.6775070873       19.1563708286
Ti          0.0563794362        8.4793616509        8.9467179417
Ti          0.0637985346        8.4539855050       19.1521334552
Ti          0.0916563818       12.2514170022        8.9407169729
Ti          0.1158856593       12.2076921920       19.1291048606
Ti          3.7571823911        0.9333130434        8.9444795369
Ti          3.7386856054        0.8918372625       19.1586663416
Ti          3.7926965165        4.7202860335        8.9412286575
```

Ti	3.7920555593	4.6847533794	19.1618026706
Ti	3.8300050868	8.4805561564	8.9489178190
Ti	3.8666398983	8.4446081290	19.1350288637
Ti	3.8563427476	12.2313301734	8.9646627837
Ti	3.8768278425	12.2209716162	19.1602455252
Ti	7.5392813905	0.9386460067	8.9420091757
Ti	7.5556550309	0.9134362883	19.1636505061
Ti	7.5790224083	4.6986300311	8.9448911231
Ti	7.6067245098	4.6715774567	19.1394764767
Ti	7.6009694465	8.4881799553	8.9637637357
Ti	7.5831857750	8.4347571603	19.1551848208
Ti	7.6135486507	12.2488057989	8.9443334140
Ti	7.5865924905	12.2105094200	19.1472655490
Ti	11.3219739946	0.9148709362	8.9449524578
Ti	11.3338759041	0.8891791944	19.1462302817
Ti	11.3445534617	4.6992552914	8.9490786321
Ti	11.3455342690	4.6627009070	19.1549642197
Ti	11.3512777468	8.4906821868	8.9325965141
Ti	11.3386061373	8.4538641732	19.1394901190
Ti	11.3794957527	12.2767041122	8.9438094645
Ti	11.3877843706	12.2362558094	19.1515724146
O	-0.0022869352	2.6967833227	3.3683648221
O	0.0529688632	2.7249890910	13.5872398290
O	0.0422433279	6.3627538345	3.3074707460
O	0.0739841164	6.3938759503	13.5275939796
O	0.0365554610	10.4888750817	3.2976670005
O	0.0701141064	10.6244989097	13.5694870377
O	0.0571622153	14.3604200491	3.3352859678
O	0.1043977842	14.2771270428	13.5746828553
O	3.7873698560	2.5819113948	3.3091705576
O	3.8091571201	2.5873940265	13.4996670280
O	3.8535913484	6.8938177594	13.5491807775
O	3.7995996168	10.5912409401	3.3357654102
O	3.8495932942	14.0203146696	3.3664805956
O	3.9398325710	13.9984399455	13.5880148648
O	7.5257450105	2.9358544584	3.2996974804
O	7.5714144631	3.0433099259	13.5023921988
O	7.5446236338	6.8164491877	3.3322298826
O	7.5521620344	6.8133286564	13.5581050817
O	7.5914860961	10.2535920603	3.3683201023
O	7.6326650070	13.9012727510	3.3109861963
O	7.6237561576	13.8264256202	13.5524590663
O	11.2926924310	3.0458418563	3.3373461330
O	11.3280291078	3.0314286238	13.5614436989
O	11.3359339155	6.4736756110	3.3680000660
O	11.3784712743	6.6566926809	13.5868645454
O	11.3810016211	10.1291967496	3.3084725621
O	11.4172219207	10.0885366363	13.5767292037
O	11.3750525260	14.2608368682	3.2952312894
O	11.4035419985	14.2923327473	13.5282467896
O	1.8920111159	0.7573902469	8.4708043919
O	1.8922493525	0.6647126339	18.6939360765
O	1.9249082065	4.5021054792	8.4152607335
O	1.9352855834	4.4660779020	18.6278358412
O	1.9296354240	8.6733374780	8.4197987329
O	1.9510197607	12.4450806147	8.4688323143
O	1.9853979708	12.4425105425	18.6518300002
O	5.6687885718	0.7042755875	8.4206872613
O	5.6874875321	0.7341865795	18.6271776142
O	5.6721856466	4.8684856876	8.4116553863
O	5.6933290932	4.5546470328	18.6279847657
O	5.6882561760	8.6694086480	8.4519524378
O	5.7130218587	8.7617654367	18.6571104211
O	5.7420228025	12.1438583761	8.4725575174
O	5.7395390181	11.9181380627	18.6747228046
O	9.4165283995	1.0630004009	8.4088330583
O	9.4386433310	0.7794805591	18.6336511748
O	9.4357685623	4.9297616176	8.4529935230
O	9.4606204410	4.9415652150	18.6651012374
O	9.4820387407	8.3694106539	8.4832545793
O	9.4699704559	8.2631871171	18.6683678861
O	9.5175864541	12.0075432038	8.4275568685
O	9.5178748023	12.0639729860	18.6205482210
O	13.1778240075	1.1409470039	8.4561034154
O	13.1922243494	1.1189141237	18.6616416377
O	13.2303598457	4.5620960393	8.4738481055
O	13.2389368515	4.5015656723	18.6823813572
O	13.2675482430	8.2774671659	8.4251400025
O	13.2832231126	8.2435251580	18.6278012167
O	13.2590673160	12.3858635902	8.4136473865
O	13.2886850751	12.1549471037	18.6276477443
O	1.6792808545	2.8313579725	5.8897982264
O	1.7309968446	2.8588078968	16.0889854042
O	1.8568224669	6.5964538101	5.8543641611
O	1.8003852451	6.6165514370	16.0573277201
O	2.1791096791	10.3480364548	5.8618907037
O	2.1855366649	10.3603530656	16.1014918145
O	2.0840118270	14.1279508357	5.9279629747
O	1.8851929926	14.1341426634	16.1084858859
O	5.5928936186	2.8218726549	5.8570279479
O	5.5340524650	2.8357268602	16.0644138647
O	5.9359836220	6.5740143227	5.8613706564
O	5.9014987863	6.5289491195	16.0566550393
O	5.8202681560	10.3558548891	5.9307551705
O	5.7395607699	10.3694001153	16.0540031146
O	5.5223635933	14.1544765572	5.8948778047
O	5.6231125457	14.2494149144	16.0644610508
O	9.6738992345	2.7968539690	5.8597515173
O	9.6647914904	2.8104661575	16.0736590811
O	9.5683812798	6.5847536109	5.9272009788
O	9.6560883606	6.6269948919	16.1073127626
O	9.2775316233	10.3808227189	5.8909912300
O	9.3542821481	10.4022764574	16.1141004770
O	9.4255389304	14.1458365807	5.8534221408
O	9.3869736546	14.1542683026	16.0655712802
O	13.3044521226	2.8064294914	5.9205765521
O	13.3154024446	2.8327434087	16.1145121399
O	13.0040279543	6.6086201058	5.8928910397
O	13.0450667595	6.6316639614	16.1096598800
O	13.1935919063	10.3724374564	5.8632602830
O	13.5327422232	14.1203244950	5.8617411981
O	13.5404510326	14.1403695445	16.1015207395
O	0.0612471592	0.9088343860	0.8050433067
O	0.1179952991	0.9213077396	11.0392238481
O	-0.2287904771	4.7083932583	0.7871239191
O	-0.2033464162	4.7212868415	10.9895752919
O	-0.1162569310	8.4873793996	0.7404316193
O	0.0857049761	8.4788041005	10.9785000249

O	0.2576586752	12.2372615705	0.7511297707
O	3.5038126666	0.9221370013	0.7898121285
O	3.5459724319	0.9467618494	10.9782941314
O	3.6138001775	4.7181584512	0.7405744675
O	3.7912290205	4.7045336809	10.9524248071
O	4.0034592404	8.4687117719	0.7523578725
O	4.0710259509	8.4581308931	10.9991293777
O	3.9296590838	12.2392499993	0.8017644026
O	3.9725554175	12.2441637859	11.0937779572
O	7.3912031098	0.9380251398	0.7416202509
O	7.5782329020	0.9409593883	10.9499206183
O	7.7435087642	4.7021350515	0.7583014693
O	7.8163881164	4.6837477924	10.9548317485
O	7.6293319436	8.4618365414	0.7979761483
O	7.7190030393	8.4839371711	11.0855558375
O	7.3477613461	12.2500754723	0.7845011408
O	7.4061904199	12.2647493439	11.0241208925
O	11.4730559807	0.9189041921	0.7590220624
O	11.5608841968	0.9063871791	10.9723721028
O	11.4119821011	4.6922973540	0.8010296263
O	11.4668237730	4.7051515940	11.0411284425
O	11.1038795762	8.4861548884	0.7808478547
O	11.1316764255	8.4880955344	10.9937760214
O	11.2120786149	12.2594068045	0.7391778996
O	11.3844318714	12.2670116707	10.9770301498
O	1.8638582388	3.0207547943	1.8050674229
O	1.9010365279	3.0698158565	11.9929414075
O	1.9021388031	6.7235187383	1.7275217999
O	1.9635541048	6.5578129882	11.9821581615
O	1.9428077732	10.1259712645	1.7715154664
O	1.9669038199	10.1853083303	12.0344792446
O	1.9621625649	14.0230583287	1.7991284932
O	2.0189117945	14.1061649542	12.0391725703
O	5.6430930598	2.9368537844	1.7215709314
O	5.6870527180	2.8314538579	11.9336483065
O	5.6851449437	6.3426539268	1.7732417658
O	5.7129075600	6.3080940781	11.9825848845
O	5.7041827906	10.2586187515	1.8004393975
O	5.7465181589	10.2958040985	12.0423500082
O	5.7161037780	14.3639895941	1.8068634268
O	5.7724607436	14.4558033101	11.9852306525
O	9.4332043265	2.5898290849	1.7752760936
O	9.4778611738	2.5835242806	11.9899370039
O	9.4512158144	6.4986543822	1.7962524164
O	9.4835479522	6.4519581189	12.0375689511
O	9.4569113089	10.5708993466	1.8051843773
O	9.5304487961	10.5797895198	12.0429086017
O	9.4867887531	14.2501072868	1.7229812289
O	9.5052332402	14.2103288138	11.9852400998
O	13.1939497223	2.7063452250	1.7978635099
O	13.2385900913	2.7214324277	12.0263046857
O	13.2001630346	6.8071779285	1.8040399520
O	13.2490668278	6.8144202341	11.9975439615
O	13.2357729435	10.4932981426	1.7239104605
O	13.2789225949	10.3810186722	11.9833955591
O	13.2817190643	13.8962747069	1.7704703815
O	13.3185803989	13.9357820521	11.9997203983
O	0.0023405852	0.8581098685	6.9160387112
O	0.0240579712	0.9804197871	17.1264332075
O	0.0073478564	4.9309257918	6.8979744431
O	0.0191832455	4.9356287295	17.1215147197
O	0.0524385114	8.5396942664	6.8526404713
O	0.0711555613	8.6813144991	17.0861304843
O	0.0927874617	12.0275570696	6.8926365997
O	0.1152465606	12.0482733232	17.0720920509
O	3.7530516096	1.1539528285	6.9005092670
O	3.7430638043	1.1324672046	17.1355796891
O	3.7929050168	4.7963548776	6.8443887482
O	3.7977847291	4.9205939605	17.0907612704
O	3.8284285266	8.2449403208	6.8865815510
O	3.8739800521	8.3109047342	17.0419938749
O	3.8490558712	12.1533916216	6.9238322625
O	3.8899655575	12.3578151791	17.0930152252
O	7.5352024976	1.0207445705	6.8462281539
O	7.5627651276	1.1661932778	17.0883930382
O	7.5796313527	4.4701244005	6.8816455903
O	7.6080529942	4.5439394644	17.0783036239
O	7.5991098634	8.4032184390	6.9208535462
O	7.5923489915	8.4129783570	17.0883755764
O	7.6081186258	12.4656181289	6.9044719295
O	7.5842099803	12.4291414330	17.0965718833
O	11.3240336491	0.6874900449	6.8811611803
O	11.3338246086	0.7696910132	17.0674170087
O	11.3406651648	4.6148385577	6.9170639229
O	11.3573660406	4.6330058237	17.1178247849
O	11.3469552369	8.7013974844	6.9067341178
O	11.3517072387	8.7086211279	17.1315670131
O	11.3785281080	12.3701449175	6.8513142558
O	11.3956047390	12.4585169455	17.0805807392
O	0.1434580193	2.8025228952	9.4640377960
O	0.1798758842	2.7683636351	19.6894656064
O	0.2565206371	6.5796710886	9.4208163149
O	0.2494094097	6.5279760806	19.6587878817
O	-0.1206131415	10.3804461429	9.4340652625
O	0.1370774878	10.3271503935	19.6114941580
O	-0.1365454287	14.1590236024	9.4680998144
O	-0.1405125437	14.1082682127	19.6464458403
O	3.9795163770	2.8006403770	9.4011406766
O	3.9530496739	2.7479319430	19.6647668379
O	3.6278635521	6.6180315224	9.4205598012
O	3.9227780074	6.5588333695	19.6112743669
O	3.6903855129	10.3861021241	9.4997885118
O	3.5690634336	10.3410430819	19.6519069714
O	3.9802308123	14.1060985019	9.4840154034
O	4.0758771198	14.0910776698	19.6848128759
O	7.3781064031	2.8295019896	9.3909574773
O	7.6628207020	2.7872793291	19.6231218751
O	7.3686832044	6.6324227358	9.4845319297
O	7.3127576975	6.5670246964	19.6418908118
O	7.6512528411	10.3523257589	9.4874769075
O	7.8256626490	10.3192172788	19.6885123477
O	7.8288570482	14.1077345453	9.4336171023
O	7.7643342934	14.0692492105	19.6488186494
O	11.1021390961	2.8372697854	9.4732389701
O	11.0756176507	2.7910347174	19.6504400070
O	11.4673951082	6.5825375871	9.4612822846
O	11.5154069419	6.5513163465	19.6807877548
O	11.5896097189	10.3531427697	9.4389267477

```
O        11.5625911202     10.3050316987     19.6476935101
O        11.2115830174     14.1511816635      9.4124177249
O        11.4897468054     14.1047127765     19.6213503834
O         1.9614642303      0.9112422076      4.3589691033
O         2.0583469239      0.9260355534     14.5744588035
O         2.1171507848      4.6867644277      4.3116423572
O         2.2069284169      4.7162402547     14.5251470198
O         1.7763225622      8.4885508888      4.2985514128
O         1.8243166417      8.5125983479     14.5019385122
O         1.7198074107     12.2692455597      4.3587443367
O         2.0539369688     12.2504583122     14.5301958908
O         5.8643644984      0.9105753275      4.3189518195
O         5.9743794412      0.9785570114     14.4792728111
O         5.5203160131      4.7122507946      4.2986798826
O         5.4794681250      4.6918805469     14.4663591118
O         5.4660110669      8.4961937608      4.3590362443
O         5.8079483820     12.2391277396      4.3633165318
O         9.2718660294      0.9360130984      4.2932103220
O         9.2561294271      0.9427390090     14.5030036346
O         9.2090334354      4.7230576019      4.3568309252
O         9.2887428500      4.7482862558     14.5689233915
O         9.5496161877      8.4655787066      4.3583869123
O         9.3636141543      8.5170923067     14.5420130997
O         9.7167654362     12.2359266140      4.3158399612
O         9.7232058377     12.2472211924     14.5238623568
O        12.9527206131      0.9411923475      4.3555941021
O        13.0335437170      0.9500167173     14.5625100685
O        13.3069610794      4.6938755115      4.3581284003
O        13.3055255218      4.7258163708     14.5586923126
O        13.4603063142      8.4641151104      4.3194203126
O        13.5657530404      8.4853110445     14.5478680125
O        13.1032510000     12.2600188022      4.3007815004
O        13.1025482216     12.2761043908     14.5281475102
O         0.3357565771     12.2484050849     10.9778758830
O         1.9718724003      8.3542439861     18.6171564662
O        13.1382837015     10.3822487651     16.0811568754
O         3.7847929249      6.7158744189      3.3009898006
F         5.7969945041     12.3426277000     14.6465267733
F         5.6637600389      8.4207634569     14.6233709491
F         3.8119959683     10.4032215442     13.5222561275
F         7.6813287364     10.3797679233     13.5249406649
    &END COORD
    &KIND O
     ELEMENT O
     MASS    15.99940
    &END KIND
    &KIND Ti
     ELEMENT Ti
     MASS    47.867
    &END KIND
    &KIND F
     ELEMENT F
     MASS    18.998
    &END KIND
   &END SUBSYS
STRESS_TENSOR ANALYTICAL
&END FORCE_EVAL
```


[S1] Burbano, M., Nadin, S., Marrocchelli, D., Salanne M. & Watson, G. W. Ceria Co–doping: Synergistic or Average Effect? *Phys. Chem. Chem. Phys.* **16**, 8230–8331 (2014).

[S2] Marrocchelli, D., Madden, P. A., Norberg, S. T. & Hull, S. Structural Disorder in Doped Zirconias, Part II: Vacancy Ordering Effects and the Conductivity Maximum. *Chem. Mater.* **23**, 1365–1373 (2011).
